# An Ensemble-Based Approach to Anomaly Detection in Marine Engine Sensor Streams for Efficient Condition Monitoring and Analysis

**DOI:** 10.3390/s20247285

**Published:** 2020-12-18

**Authors:** Donghyun Kim, Sangbong Lee, Jihwan Lee

**Affiliations:** 1 Korea Marine Equipment Research Institute, Busan 49111, Korea; kimdonghyun9942@gmail.com; 2 Lab021 Shipping Analytics, Busan 48508, Korea; sblee@lab021.co.kr; 3 Department of Industrial Data Engineering, Industrial Data Science and Engineering, Pukyong National University, Busan 48513, Korea

**Keywords:** marine engine, two-stroke diesel engine, onboard sensor, condition monitoring, unsupervised anomaly detection, ensemble learning, clustering analysis, anomaly analysis

## Abstract

This study proposes an unsupervised anomaly detection method using sensor streams from the marine engine to detect the anomalous system behavior, which may be a possible sign of system failure. Previous works on marine engine anomaly detection proposed a clustering-based or statistical control chart-based approach that is unstable according to the choice of hyperparameters, or cannot fit well to the high-dimensional dataset. As a remedy to this limitation, this study adopts an ensemble-based approach to anomaly detection. The idea is to train several anomaly detectors with varying hyperparameters in parallel and then combine its result in the anomaly detection phase. Because the anomaly is detected by the combination of different detectors, it is robust to the choice of hyperparameters without loss of accuracy. To demonstrate our methodology, an actual dataset obtained from a 200,000-ton cargo vessel from a Korean shipping company that uses two-stroke diesel engine is analyzed. As a result, anomalies were successfully detected from the high-dimensional and large-scale dataset. After detecting the anomaly, clustering analysis was conducted to the anomalous observation to examine anomaly patterns. By investigating each cluster’s feature distribution, several common patterns of abnormal behavior were successfully visualized. Although we analyzed the data from two-stroke diesel engine, our method can be applied to various types of marine engine.

## 1. Introduction

The main engine is the most important subsystem that provides the propulsion power of the vessel. Because the failure of the engine during the operation may cause a tremendous economic loss [1], the maintenance of the engine is considered as a critical activity, not for the maintenance routine, but for the vessel classification, which is a process that verifies equipment against a set of technical standards [2]. In maritime industry, the standard practice of engine maintenance follows Planned Maintenance System (PMS), where the machinery is replaced at predetermined time intervals or operating hours, regardless of its actual status [3]. From the economic point of view, however, PMS may not be an optimal strategy because it may have an unnecessary substitution of the machinery. 

An alternative strategy to PMS is Conditioned-based Maintenance (CM), wherein the maintenance is carried out based on the condition of the machinery, which is detected by measuring several parameters during the vessel operation [4]. Fortunately, the recent development of IT technology has enabled real-time access to the machinery’s condition and energy efficiency using data collected from onboard sensors [5,6,7,8,9,10,11,12,13]. Such sensor-based monitoring can be used to detect abnormal behavior of the system that may indicate the degradation or fault of the system. This study proposes a data-driven approach that enables conditioned-based monitoring of the vessel’s main engine utilizing a machine learning algorithm. 

We adopt an unsupervised approach in the detection of anomalous system behavior. Although most industries, including the maritime industry, continuously collect data from sensors, most of the data usually comes from the normal operating condition, and a comprehensive fault dataset is usually hard to obtain. Thus, anomaly detected from our methodology may not be directly related to the system fault because any behavior that shows a large deviation from the normal dataset can be detected as an anomaly. However, they could be used for the initial screening of engine status monitoring and can be combined with further analysis, including fault isolation and diagnosis. 

Previously, several unsupervised methods have been proposed to analyze onboard sensor data in anomaly detection for the marine engine. The clustering-based approach adopts a clustering algorithm to identify clusters in the sensor data first and then check whether new data belongs to existing clusters. They assume that normal instances have stronger adherence to clusters than the anomaly. Perera and Mo [14,15] classified the most frequent operating regions of the marine engine. In their work, the Gaussian Mixture Model is adopted to represent the cluster of operating regions as a mixture of probability distributions. Brandsæter and Venem [16] proposes an efficient online method to calculate the degree of abnormality from clusters. Vanem and Brandsæter [17] compare several clustering algorithms by analyzing anomaly detection results. Although the Clustering-based approaches are intuitive and easy-to-implement, it suffers from unstable because the clustering result may be largely affected by the number of clusters, which should be specified by the user. Although there are several guidelines for determining the good cluster numbers, they cannot be applicable to the unsupervised dataset. As an alternative approach, Bae et al. [18] proposed a Statistical Process Control (SPC)-based approach to the anomaly detection of the vessel engine. To remove the distributional assumption of the dataset, a Bootstrap-based T2 multivariate chart proposed by Phaladiganon et al. [19], is adopted to determine the threshold for each sensor data. If one of the sensor values is out of the threshold, the data point is detected as an outlier. However, SPC-based approach suffers from low performance when the dataset involves high dimensional spaces [20]. 

To overcome the limitation of the above approaches, we propose an ensemble-based anomaly detection method to operate on a large-scale high-dimensional dataset. The idea is to apply several algorithms with varying hyperparameters to the same dataset and then combine each classifier’s anomaly detection result. Because the anomaly is determined by the combination of multiple classifiers, the result is robust to the choice of hyperparameters without loss of its prediction power. To demonstrate our methodology, a data stream obtained from a 200,000-ton bulk cargo ship operated by a Korean shipping company that is collected during ten months are analyzed. The data set consists of comprehensive parameters representing engine performance, including engine rotation per minute (RPM), temperature, and pressures of lubricant oil and cooling waters. Several preprocessing steps were conducted to reduce the data size and select the informative sensor parameters. The ensemble-based algorithm then trains the preprocessed data to detect the anomalies from the input data. After detecting the anomaly, clustering analysis was conducted to the anomalous observation to examine anomaly patterns. By investigating each cluster’s feature distribution, several common patterns of abnormal behavior were successfully visualized. The result shows that the proposed method can be successfully applied to the large and high-dimensional sensor streams. Although we analyzed the data from two-stroke diesel engine, our method can be applied to various types of marine engine. 

The remainder of this paper is organized as follows. Section 2 explains about the target vessel and data set collected from the vessel. Section 3 addresses the procedures used for data preprocessing. Section 4 illustrates the ensemble-based machine learning model used for anomaly detection of the engine status. In Section 5, an in-depth discussion about anomaly detection results was performed. Finally, Section 6 addresses the limitation and future work of this study. 

## 2. Data Description

**Figure 1:**
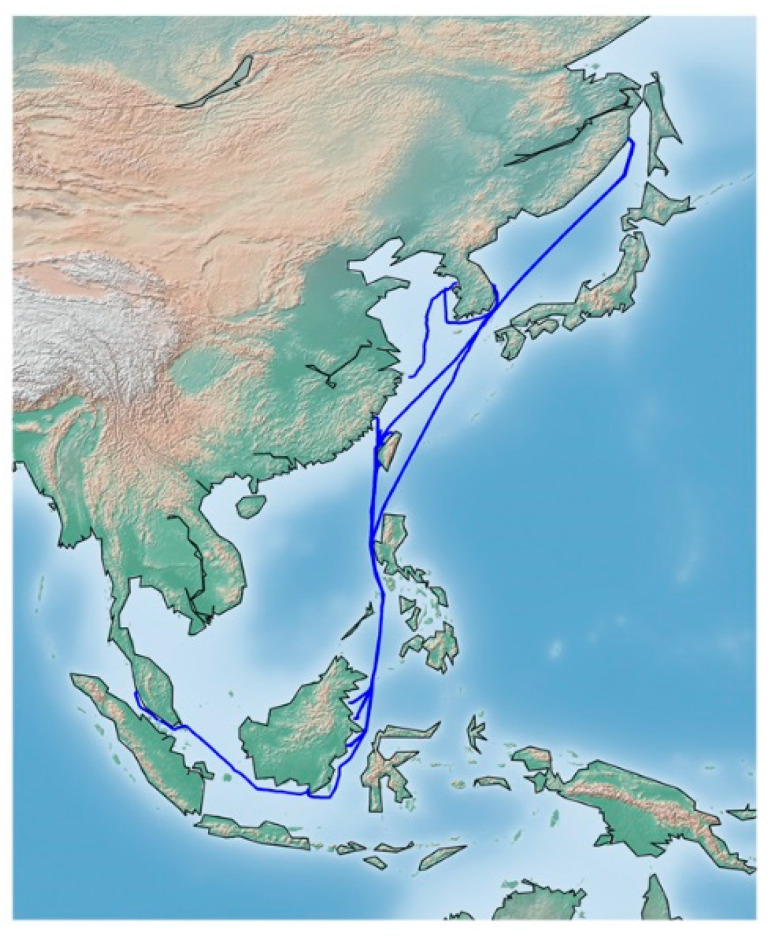
Vessel operation routes over the data collection period.

This section explains the description of our target vessel and the dataset used in anomaly detection. The target vessel is a 200,000-ton bulk cargo ship, and its detailed specification is in Table 1. The data collection period spans about ten months, starting from 2019 July to 2020 April. As shown in Figure 1, its routes include main ports in Asian countries including Korea, Russia, Singapore, and Taiwan. The sensor measured the data at a one-second interval, resulting in a total of 22,513,800 observations. 

The engine model used in the vessel is MAN B&W MC50, which is a slow-speed two-stroke engine [21]. The engine adopted Variable Injection Timing (VIT) systems that control the timing of the start of the fuel injection. The coolant system uses lubricant cooling for the rotating part (Crankshaft, Piston), and the fixed part (Cylinder Head, Jacket) is cooled with fresh water. The coolant is cooled by seawater in a separate heat exchanger.

In the raw dataset, more than 150 data streams were collected by onboard sensors. Some parameters were related with navigational information such as Global Positioning System (GPS) location, ground speed, wind speed, water level, etc. In contrast, others were related with subsystems’ status, including engine, generator, thruster, and cargo management system, etc. In this study, we only included parameters that are attached on main engine subsystems. Other parameters that come from other subsystems were excluded because they are not in our interest. As a result, the chosen parameters are shown in Table 2. 

## 3. Data Preprocessing

**Figure 2:**
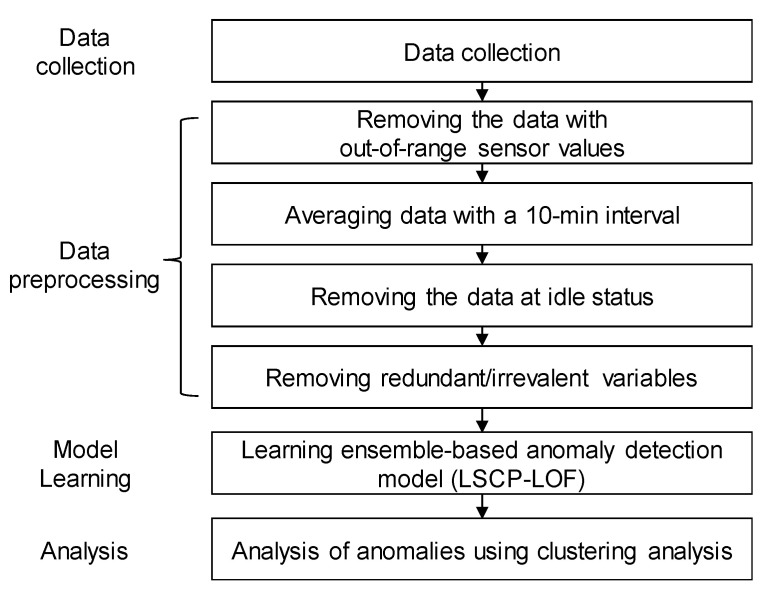
The overall framework for vessel main engine anomaly detection.

All the data-driven approach requires representative training dataset. However, the raw data stream is not complete. It may contain out-of-range values, missing values, redundant variables, and irreverent information. If the raw data is not carefully screened, then the resulting model will not perform well on the new data. Thus, in this study, several preprocessing methods were applied to improve the quality of the dataset. The overall analysis framework is shown in Figure 2.

First, out-of-range values that exceed the acceptable sensor value ranges were removed. In some cases, sensor value shows zero or extremely large values that are out of acceptable range of sensors. Those values are usually the consequence of signal loss either from the sensors or from the communication. Because both cases were not related to the failure of the engine, it is natural to remove such outliers in the training data set. 

Then, we reduce the dataset by averaging its value with a 10-min interval. One reason for this transformation is that the current data set (measured with a second interval) is too huge to train the model. Besides, the vessel engine usually undergoes slow changes during the operation compared to other vehicles such as the car or the airplane. Thus, averaging the dataset with a 10-min interval may be enough for training the model. As a result, the size of the dataset was reduced to 37,523.

**Figure 3:**
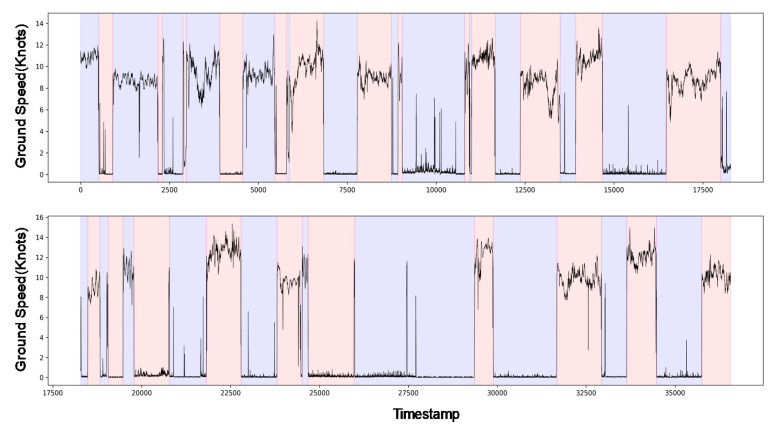
Time series of the vessel ground speed. Moreover, the windows-based change detection method is also applied to find operational regions among the dataset. The alternating color region indicates whether the vessel operates or not.

Next, we exclude the data collected when the vessel was idle because the vessel engine does not operate during that period. As shown in Figure 3, the vessel shows the alternating operational status (idle and normal operation) during the data collection period. This study adopts a window-based change point detection algorithm to the ground speed time-series data to distinguish the vessel’s operational status. The algorithm tries to detect the rapid change points using two windows, which slide along the data stream. The statistical properties of each window are compared with a discrepancy measure. For a given cost function c(⋅), a discrepancy measure d(⋅,⋅) as follow:

(1) $$d(y_{u,v},y_{v,w})=c(y_{u,w})-c(y_{u,v})-c(y_{v,w})$$

where $y_{t}$ is the input time series at time point *t* and u<v<w are indexes. If the discrepancy measure between two sliding windows is smaller, this indicates that there is no change point at v. On the other hand, if the sliding windows fall into two dissimilar segments, the discrepancy is significantly higher, suggesting that v is a change point. In this study, such a change point indicates the boundary between the operational status of the vessel. Because the time window is considered for change point detection, this method is less sensitive to the noise data. For more details about the methods, please refer to [22]. Figure 3 also shows change points detected by the time-window based method. In this study, we consider the area whose average ground speed is over 6 knots. Further, according to an expert opinion, we determined to consider the dataset whose RPM value is over 70. 

**Figure 4:**
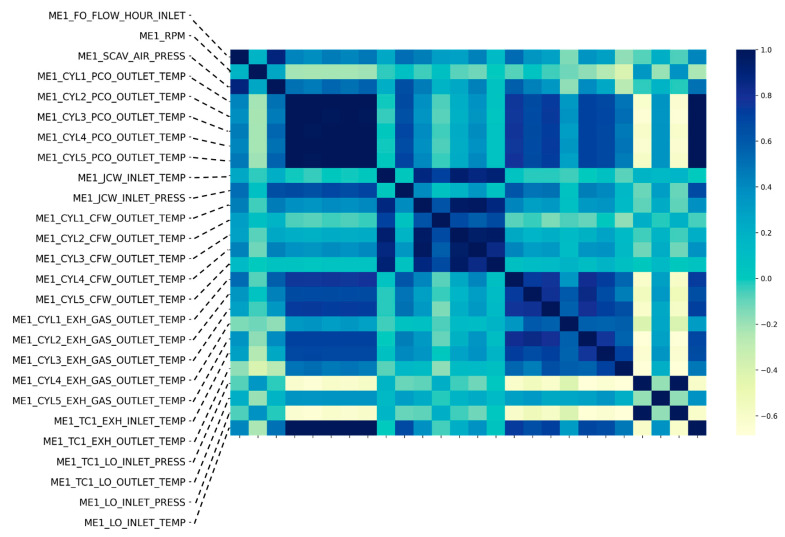
Correlation heatmap among entire feature set.

Then, feature selection and transformation was conducted. The feature selection result and was summarized in Table 3. As shown in the table, from the original dataset, some parameters (ME1 RPM ECC, ME1 SCAV AIR PRESS ECC) were obtained from duplicated sensors of other sensors (ME1 RPM, ME1 SCAV AIR PRESS) in case of sensor failures. Because parameter values of original and duplicated sensors were exactly same throughout the data collection period, we excluded duplicated sensor parameters from the dataset. In addition, parameters related with the fuel status (ME1 FO FLOW INLET, ME1 FO DESNITY INLET, ME1 FO TEMP INLET, ME1 FO TOTALIZER INLET) were removed. Of course, the change in fuel density or temperature may affect the performance of the engine. Especially, if a vessel sailed through an emission control area, such as the western part of the United States that regulates the use of low sulfur oil, the reduced lubrication effect due to low-sulfur oil might increase the probability of accidents, such as piston sticking. However, because our vessel has not sailed through an emission control area, there was no significant change in fuel oil (such as viscosity) during the data collection period. Moreover, the vessel used fuel additives to prevent the problem that may arise from fuel status. Finally, instead of using individual sensor value of individual cylinder, we use averaged value because sensor values from five cylinders show high correlation with each other as shown in Figure 4. As a result, the variable size was reduced from 32 to 14. 

We may use more sophisticated variable reduction techniques such as Principal Component Analysis (PCA) or Independent Component Analysis (ICA) to reduce the variables further. However, we determine to preserve the current variables to utilize them in several analyses after detecting anomalies for examining common patterns and causes for anomalies. As a result, the comparison between dataset and preprocessed dataset is shown in Table 4.

## 4. Ensemble-Based Method for Anomaly Detection

This section outlines the anomaly detection algorithm applied to the preprocessed dataset. This study adopts an unsupervised approach because we have no labeled dataset about the failure of the vessel’s main engine during the data collection period. The unsupervised approach assumes that all the training data shows the normal condition [23]. Thus, if a new observation shows a large deviation from the training set, it is considered an anomaly. Unsupervised anomaly detection may show poor performance if the distribution of the normal dataset is heavy-tailed, or the normal data point is too centered to mimic the anomalous data point [24]. Moreover, the low-accuracy problem may be more severe when the feature space of the dataset is high-dimensional [23]. 

To remedy this problem, ensemble learning is also applied to model learning. The ensemble approach, which combines multiple base estimators in anomaly detection, is considered as a strategy to improve the model accuracy and stability because one can reduce the effect of variance on modeling accuracy by running the model multiple times [25]. Ensemble approach can be categorized as model-centric when multiple based estimators of the different hyperparameter are combined to predict anomaly score, while it is categorized as the data-centric when the different derivatives of the dataset are applied to the same model. Several ensemble-based approaches, such as feature bagging, parametric ensemble, and sub samplings, are available. 

### 4.1. Base Anomaly Detection Algorithm: Local Outlier Factor

In this study, the Local Outlier Factor (LOF) [26] is applied as a basic anomaly detector. LOF is considered as an instance-based method because it firstly finds a relevant instance of the training data and makes a prediction using the information of these instances. Because this approach does not require the design of generic models, it is often referred to as memory-based methods. LOF [17] and the k-nearest neighborhood-based method [27] was its successful implementation. One of the problems in instance-based methods is that the performance of the anomaly detection may be severely affected by the local distributions of the data. LOF addresses this problem by using density information of its neighborhood point. For a given data point $x_{i}$, let $D^{k}(x_{i})$ be the distance between $x_{i}$ and its k-nearest neighbor, and $L_{k}(x_{i})$ be the set of points within the k-nearest neighbor distance. Then, we calculate the reachability distance between two data points $x_{i}$ and $x_{j}$ $R_{k}(x_{i},x_{j})$ is calculated as follows:

(2) $$R_{k}(x_{i},x_{j})=\max\{dist(x_{i},x_{j}),D^{k}(x_{j})\}$$

when *j* is in a dense region and the $x_{i}$ is far from $x_{j}$, the reachability index will be equal to the true distance. If j is in sparse region, on the other hand, the reachability index will be smoothed out by its k-nearest neighbor distance. In this way, we can calculate the average reachability distance $AR^{k}(x_{i})$ of $x_{i}$ by averaging reachability distance of its k-nearest neighborhood points:

(3) $$AR^{k}(x_{i})=MEAN_{j\in L_{k}(x_{i})}R_{k}(x_{i},x_{j})$$

the local outlier factor is the average ratio of $AR^{k}(x_{i})$ with respect to its k-nearest neighborhood of $x_{i}$:

(4) $$LOF_{k}(x_{i})=MEAN_{y_{i}\in L_{k}(x_{i})}\frac{AR_{k}(x_{i})}{AR_{k}(x_{j})}$$

As the LOF algorithm can detect “local” outliers regardless of the data distribution of normal behavior, it has been applied to various applications, including network intrusion detection and process monitoring [28]. Due to the computational complexity of the LOF algorithm, however, its application to large data with high dimension has been limited. This issue can be more critical for real-time application systems.

### 4.2. Ensemble-Based Approach to Anomaly Detection: LSCP

As an ensemble approach to anomaly detection, Locally Selective Combination in Parallel Outlier Ensembles (LSCP), which is proposed by Zhao et al. [29], is adopted. LSCP is proposed to solve the local data problem when the data consists of heterogeneous distribution, thus cannot be represented by the one generic model. The presence of the local data structure, thus, is considered as one of the main causes that lower the performance of the unsupervised anomaly detection algorithm. LSCP tries to solve this problem by identifying local regions obtained from its nearest neighbor and building competitive ensemble detectors for each local region, thus, providing more robust predictions. Moreover, LSCP utilizes a feature bagging strategy to cope with the problem arise in high-dimensional feature space. 

**Figure 5:**
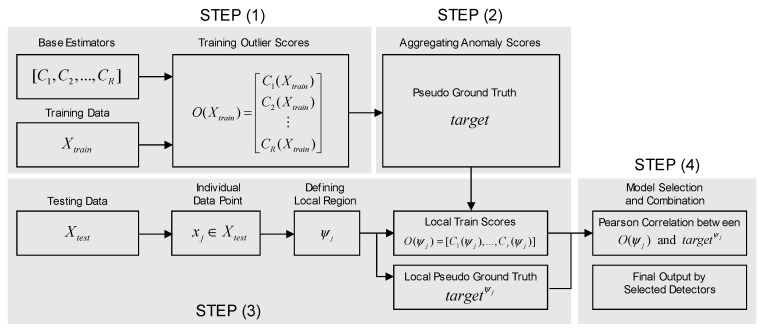
Locally Selective Combination in Parallel Outlier Ensembles (LSCP) Procedure (adopted from Zhao et al. [22]).

LSCP consists of four major steps, as shown in Figure 5. In the first stage, generate pseudo-ground truth labels are generated from the ensemble. Let $X_{train}$ be training data, and $C=\{C_{1},C_{2},\ldots ,C_{R}\}$ be a collection of base detectors with different hyperparameter settings. Moreover, let $O(X_{train})$ be the matrix of the anomaly score $O(X_{train})=[C_{1}(X_{train}),\ldots ,C_{r}(X_{train})]$. Then, the pseudo ground truth denoted by the *target* is obtained by score aggregation of base estimators *C* as follows:

(5) $$target=f(O(X_{train}))$$

In the second stage, local region is constructed. Given a data instance to test $x_{j}$, the local region $\psi_{j}$ is defined as its k-nearest neighborhood defined as the follows:

(6) $$\psi_{j}=\{x_{i}|x_{i}\in X_{train},x_{i}\in L_{k,ENS}(x_{j})\}$$

To define the local region $L_{k,ENS}(x_{j})$, t groups of [d/2, d] features are randomly selected and k nearest data object is identified. $x_{i}$ is included in $L_{k,ENS}(x_{j})$ when $x_{i}$ are included in the neighborhood more than t/2 times.

In the Third stage, model selection and combination are conducted. For testing instance $x_{j}$, let $target^{\psi_{j}}$ be the pseudo ground target value from its k nearest neighborhood:

(7) $$target^{\psi_{j}}=\{target_{x_{i}}|x_{i}\in \psi_{j}\}$$

(8) $$O(\psi_{j})=[C_{1}(\psi_{j}),\ldots ,C_{r}(\psi_{j})]$$

Moreover, let $O(\psi_{j})$ be the training score matrix retrieved from its anomaly score matrix:

Then, the correlation between each base detector and pseudo ground truth over the local region is calculated. Pearson correlation is applied between $target^{\psi_{j}}$ and $O(\psi_{j})$. 

In the final step, a histogram of the Pearson correlation score of each detector is constructed, and then binned with b equal intervals. Then, the collection of detectors belonging to the most frequent intervals are kept for the ensemble for the later stage. Finally, selected detectors scores are combined with the average of maximum strategy. In Zhao et al. [29], LSCP shows better performance on many real datasets. LSCP is also considered in this study because our vessel dataset is collected from several heterogeneous routes, indicating the presence of local structures.

## 5. Experimental Result 

### 5.1. Anomalies Detection Result

This section illustrates the result of the anomaly detection analysis. To make an ensemble anomaly detector, we combined 30 different LOF detectors. To enhance the robustness of an ensemble detector, it is required to ensure the diversity of base detectors by setting different hyperparameters. In the case of LOF, the dominant hyperparameter is k, which is the number of nearest neighbors to consider. Thus, 30 different hyperparameter set is randomly drawn from integer intervals ranging from 5 to 150. The numerical experiment was performed on Python 3.6. We used the PYOD (python toolkit for detection of outlying objects) in the implementation of LSCP [30]. The computing environment was CPU 2.2 Ghz, RAM 13 Gb.

**Figure 6:**
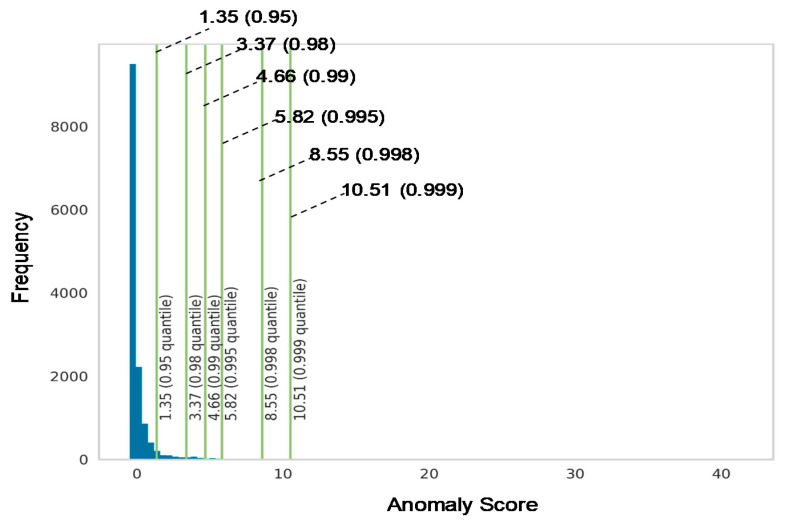
Histogram of anomalies score.

Figure 6 shows the histogram of the anomaly scores obtained from the LSCP algorithm. The vertical line indicates the anomaly thresholds with different percentile values. Because the histogram has a very thin tail part, it seems that the anomalous data object is well separated from the normal dataset. As shown in Figure 6, we further highlighted the tail part by limiting the y-axis value to determine the proper threshold. In this study, percentile 0.998 is considered as the threshold because there is a small inflection of histogram, which may suggest the separation between different groups. 

**Figure 7:**
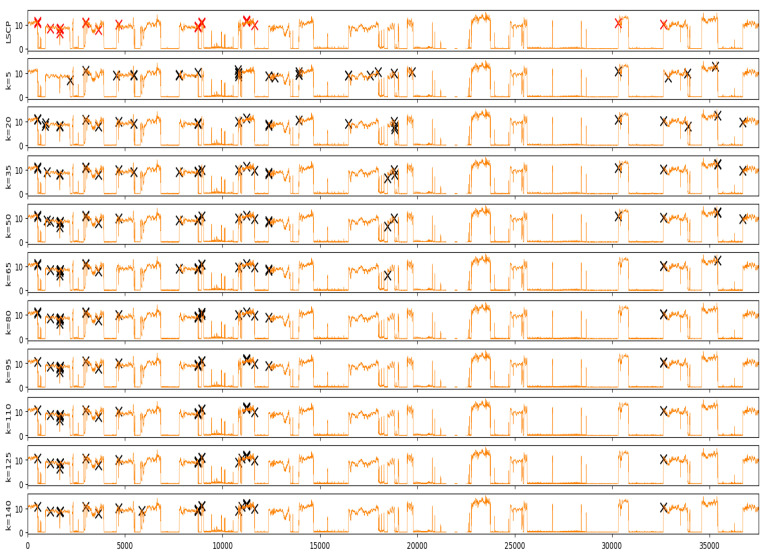
Comparison of anomaly detection results. The red mark indicates anomalies detected by the ensemble-based approach (LSCP), while the black mark indicates anomalies from individual detector Local Outlier Factor (LOF).

In Figure 7, we compared the anomalies obtained from our ensemble-based model (LSCP) with several individual detectors (LOFs) of varying hyperparameters. As shown in the figure, the anomaly detection result of an individual detector varies according to the hyperparameter. This result suggests that depending only on a single anomaly detector may be biased with the local data structure. On the other hand, anomalies detected from the ensemble-based method seems to be more robust because it includes data points that commonly appear across individual detectors. 

### 5.2. Anomalous Pattern Identification Using Clustering Analysis

**Figure 8:**
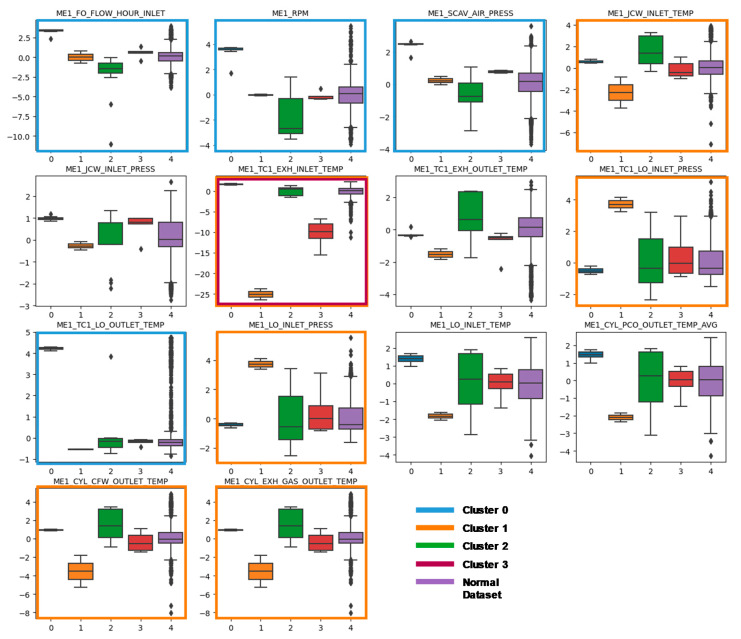
Comparison of boxplots among anomalous data cluster. Clusters that show large deviation from other groups are highlighted by the cluster colors.

We identified clusters of an anomalous dataset to examine the typical patterns of anomalous engine behavior. To this, we applied the K-means algorithm to the anomalous data points detected from our ensemble-based algorithm. As a result, we found four clusters of anomalous engine behavior. Figure 8 compares the distribution of entire variables of each anomalous data cluster. For each sensor variable, we = highlighted the cluster that shows a large deviation from the distribution of normal data points. As shown in the figure, most of the variables were highlighted by cluster 0 (highlighted by the blue line) or cluster 1 (highlighted by the green line). However, cluster 2 and 3 show little distinction from the normal data distribution. Table 5 summarizes the anomalous features associated with each cluster.

Anomalies detected from Cluster 0 show a high value in fuel oil flow rate, RPM, scavenging air pressure, and turbocharger lubricant oil temperature. A possible cause of this anomaly may be the engine’s acceleration because all of the relevant parameters seem to be the result caused by the engine acceleration [21]. On the other hand, the possible explanation about the anomalous parameters in cluster 1 seems to engine overcooling of the engine, wherein the normal temperature at which the engine operates cannot be reached. Because the engine overcooling also can damage an engine just as overheating, this region requires further investigation [31]. Cluster 3 is almost the same as cluster 0 except for lower turbocharger exhaust gas temperature. One of the possible causes of this anomaly may be the abnormal intake airflow in a marine engine [32]. 

### 5.3. Anomalous Engine Status Analysis with Vessel Operational Information

**Figure 9:**
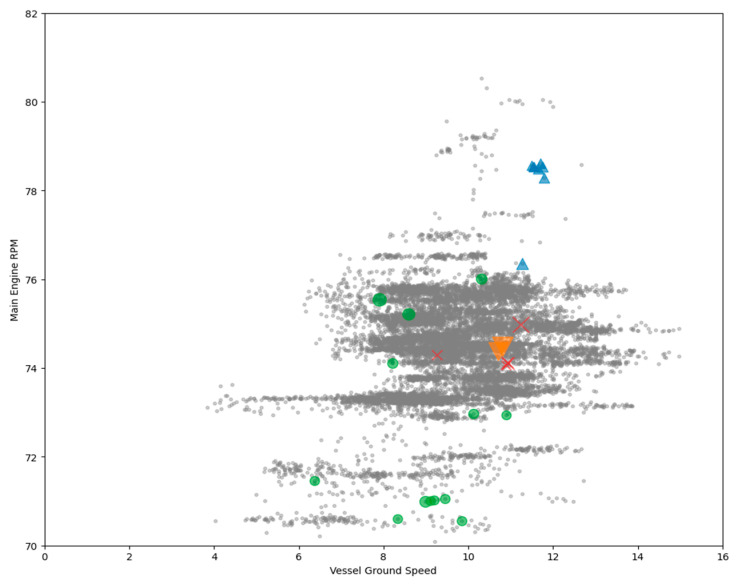
Anomalies plotted over speed vs. RPM scatter plot.

We conducted several analyses to find some potential causes of the anomalous data point. First, we analyzed the anomalous data point by examining its location on the vessel speed vs. power curve, as shown in Figure 9. Usually, there is a positive relationship between the rpm of the engine and the vessel’s ground speed. If most of the anomalous data points have high speed and RPM value, then the engine’s load required to operate the vessel at high speed may cause the anomaly. However, as the figure suggests, except for cluster 0, the anomalous data point in other clusters seems to spread over the speed vs. power curve. This result suggests that our anomalous data points may not have a single cause and calls for further investigation. 

**Figure 10:**
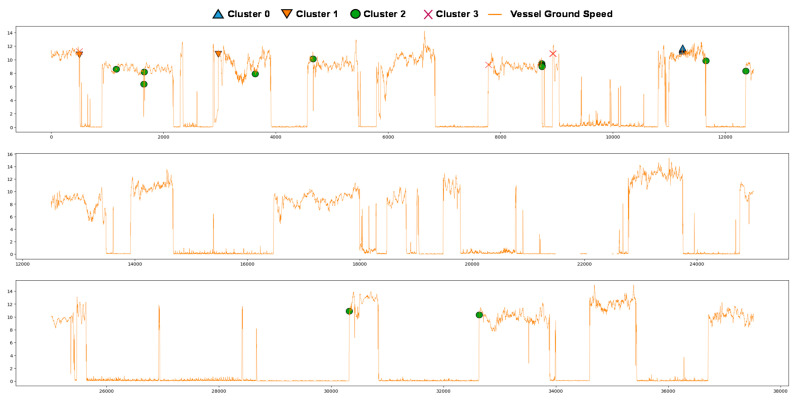
Anomalies plotted over the ground speed of the vessel.

We also examined the anomalous data point and the time series of ground speed, as shown in Figure 10. As the figure indicated, most of the data points in cluster 1, cluster 2, and cluster 3 involved the rapid vessel speed change. This result suggests that the anomaly may be related to the acceleration or deacceleration, which may cause damage to the engine.

**Figure 11:**
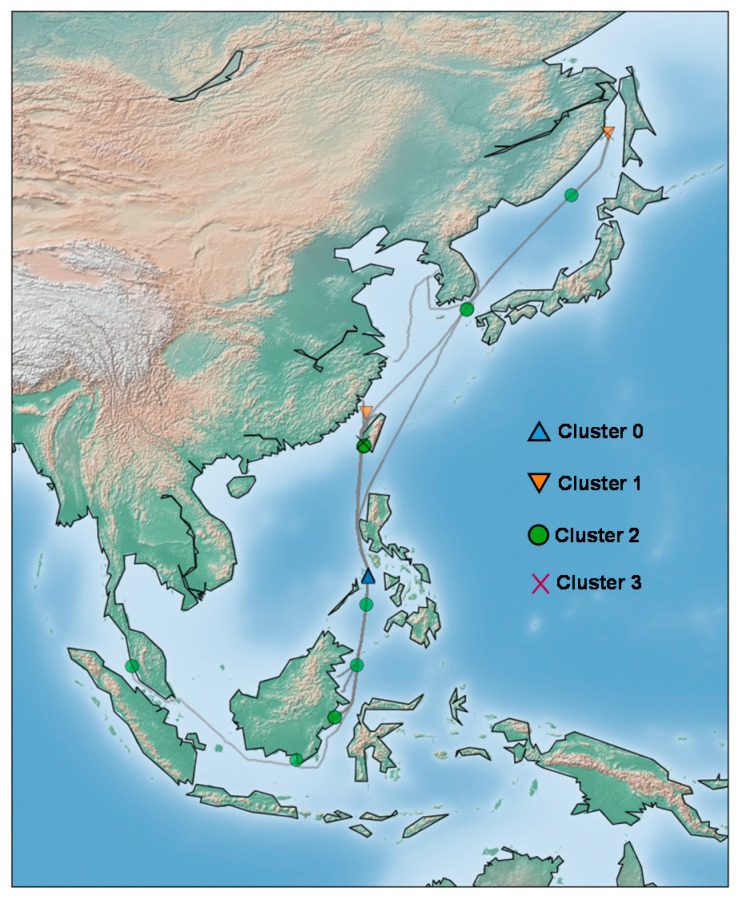
Anomalies plotted over the vessel routes.

Finally, we plotted the anomaly over the vessel route, as shown in Figure 11. The thin black line illustrates the navigation route of the ship for over ten months. As shown in this figure, most of the anomalies occurred in near lands, except few cases. The possible explanation for this might be that the vessel is usually driven at a low speed in the coastal waters to prevent an accident, and the engine is operated in a different pattern than usual due to frequent changes in speed. For this reason, data in the coastal waters can be classified as anomalous. We think that such information will help locate the cause of engine anomaly during ship operation in the future. 

## 6. Conclusions

In this work, a machine learning approach is adopted to detect the anomalous vessel main engine. We collected an actual dataset of a large-scale bulk carrier over ten months. This study adopted an ensemble-based algorithm to learn the large-scaled and high-dimensional engine sensor streams. As a result, each data point was successfully measured with a unified measure. With this framework, one can detect the anomalous engine behavior that shows a large deviation from the normal condition. In this study, we also conducted a clustering analysis to examine the common patterns of anomalies and which can provide information for the engine diagnosis.

The limitations of our research are as follows. First, the current dataset does not include external factors, such as the seawater temperature or air temperature. Although the cooling system in our target vessel operates to control the effect of such external factors, the relationship between external factors and engine performance should be investigated in future studies. Moreover, more rigorous preprocessing may improve the analysis result. Currently, only the outlier whose sensor value is outside of measurement range were removed from the dataset. However, some outliers may be within the measurement range, but does not satisfy the physical constraint. For example, the temperature of the exhaust gases at the inlet of the turbocharger must be higher than at the outlet. Accommodating such physical condition across sensor variables could be considered in the future works. Moreover, in future studies, we need to obtain a complete dataset. We need to increase the dataset size by extending the data collection period or combining another vessel’s dataset with the same engine type.

Despite the above limitation, the unsupervised approach proposed in this paper could be used for the initial screening of the engine status monitoring and can be combined with other fault diagnosis methods. One of the natural extensions of this work is to apply an existing failure mode analysis framework to the anomalies detected by our data-driven approach. There are several works for identifying possible cause and symptoms of marine engines. Those failure modes were usually obtained from the expert knowledge [33] or the simulation experiment [32]. The development of the framework and visualization scheme for relating such failure modes and the anomalies may be helpful for fault isolation and diagnosis. Our methodology can also be extended to another subsystem. The current work only analyzed the engine-related parameters. However, the modern vessel collects sensor stream from various subsystems including cargo management, or power generation system. Considering those subsystems, thus, would be a fruitful area for future works. Another possible extension of this work is to develop more efficient method for analyzing anomalies patterns. Even though clustering analysis was conducted to explain the common cause of anomalous data, it still depends on the visual inspection, making it difficult to explain the cause of anomaly quantitatively. We can improve the analysis by adopting an Explainable Artificial intelligence (EAI) framework, such as Shapley Additive Explanation (SHAP) [34] or Local Interpretable Model-agnostic Explanations (LIME) [35], which quantifies feature contribution to an individual anomalous data point. With feature importance information, more rigorous analysis for categorizing anomalous patterns by focusing on problematic sensor values may be possible.

## Figures and Tables

**Table 1:** Specification of the target vessel.

Specification	
Length Overall Length between perpendiculars Breadth Depth Draught Deadweight	269.36 m 259.00 m 43.00 m 23.80 m 17.3 m 152.517 metric t

**Table 2:** Description of parameters.

Sensor Name	Description
ME1 FO FLOW HOUR INLET	The consumption rate of fuel oil
ME1 FO TOTALIZER INLET	Cumulative consumption of fuel oil
ME1 FO TEMP INLET	The temperature of fuel oil
ME1 FO DENSITY INLET	The density of fuel oil
ME1 RPM ECC	Engine rotation per minute (RPM)
ME1 RPM	Same as above
ME1 SCAV AIR PRESS ECC	The Pressure of scavenging air
ME1 SCAV AIR PREE	Same as above
ME1 FO INLET TEMP	The inlet temperature of fuel oil
ME1 FO INLET PRESS	Inlet pressure of fuel oil
ME1 CYL1 PCO OUTLET TEMP ME1 CYL2 PCO OUTLET TEMP ME1 CYL3 PCO OUTLET TEMP ME1 CYL4 PCO OUTLET TEMP ME1 CYL5 PCO OUTLET TEMP	The outlet temperature of cylinder piston cooling oil
ME1 JCW INLET TEMP	The inlet temperature of jacket cooling water
ME1 JCW INLET OUTLET	The outlet temperature of jacket cooling water
ME1 CYL1 CFW OUT TEMP ME1 CYL2 CFW OUT TEMP ME1 CYL3 CFW OUT TEMP ME1 CYL4 CFW OUT TEMP ME1 CYL5 CFW OUT TEMP	The outlet temperature of cylinder block cooling water
ME1 CYL1 EXH GAS OUTLET TEMP ME1 CYL2 EXH GAS OUTLET TEMP ME1 CYL3 EXH GAS OUTLET TEMP ME1 CYL4 EXH GAS OUTLET TEMP ME1 CYL5 EXH GAS OUTLET TEMP	The outlet temperature of exhaust gas
ME1 TC1 EXH INLET TEMP	The inlet temperature of exhaust gas of turbocharger
ME1 TC1 EXH OUTLET TEMP	The outlet temperature of exhaust gas of turbocharger
ME1 TC LO OUTLET TEMP	The outlet temperature of lubricant oil
ME1 LO INLET PRESS	Inlet pressure of lubricant oil
ME1 LO INLET TEMP	The inlet temperature of lubricant oil

**Table 3:** Removed Parameters and Reason.

Reason	Parameters
Fuel Oil Status Indicator (not affect engine condition)	ME1 FO FLOW HOUR INLET, ME1 FO DENSITY INLET, ME1 FO TEMP INLET, ME1 FO TOTALIZER INLE
Duplicated sensors	ME1 RPM ECC, ME1 SCAV AIR PREES ECC
Aggregate value by averaging	ME1 [CYL1~CYL5] PCO OUTLET TEMP ME1 [CYL1~CYL5] PCO OUTLET TEMP ME1 [CYL1~CYL5] CFW OUTLET TEMP

**Table 4:** Comparison between original dataset and preprocessed dataset.

	Original Dataset	Preprocessed Dataset
Parameters	ME1 FO FLOW HOUR INLET ME1 FO TOTALIZER INLET ME1 FO TEMP INLET ME1 FO DENSITY INLET ME1 RPM ECC ME1 RPM ME1 SCAV AIR PRESS ECC ME1 SCAV AIR PRESS ME1 FO INLET TEMP ME1 FO INLET PRESS ME1 [CYL1~CYL5] PCO OUTLET TEMP ME1 JCW INLET TEMP ME1 JCW INLET OUTLET ME1 [CYL1~CYL5] CFW OUT TEMP ME1 [CYL1~CYL5] EXH GAS OUTLET TEMP ME1 TC1 EXH INLET TEMP ME1 TC1 EXH OUTLET TEMP ME1 TC LO OUTLET TEMP ME1 LO INLET PRESS ME1 LO INLET TEMP	ME1 RPM ME1 SCAV AIR PRESS ME1 FO INLET TEMP ME1 FO INLET PRESS ME1 CYL PCO OUTLET TEMP (Average value of 5 cylinders) ME1 JCW INLET TEMP ME1 JCW INLET OUTLET ME1 CYL CFW OUT TEMP (Average value of 5 cylinders) ME1 CYL EXH GAS OUTLET TEMP (Average value of 5 cylinders) ME1 TC1 EXH INLET TEMP ME1 TC1 EXH OUTLET TEMP ME1 TC LO OUTLET TEMP ME1 TC LO INLET TEMP ME1 TC LO INLET PRESS
Number of Observations	22,513,800 (one second interval)	37,523 (ten minutes averaging)

**Table 5:** Anomalous Parameters of Each Cluster.

Clusters	Anomalous Features
Cluster 0	High fuel oil flow rate High engine RPM High scavenging air pressure High turbocharger lubricant oil outlet temperature
Cluster 1	Low jacket cooling water inlet temperature Low turbocharger exhaust gas inlet temperature High turbocharger lubricant oil inlet pressure High lubricant oil inlet temperature Low cylinder block cooling water temperature Low cylinder exhaust gas outlet temperature
Cluster 3	High scavenging air pressure Low turbocharger exhaust gas inlet temperature
